# Severe paracoccidioidomycosis, with a fatal outcome and incidence related to an environmental event^☆^

**DOI:** 10.1016/j.abd.2021.12.006

**Published:** 2022-10-14

**Authors:** Fernanda Altoé Stringuini, Priscila Oliveira Naback, Luciana Ferreira Araújo, Ricardo Barbosa Lima, Carlos José Martins

**Affiliations:** Service of Dermatology, Hospital Universitário Gaffrée e Guinle, School of Medicine and Surgery, Universidade Federal do Estado do Rio de Janeiro, Rio de Janeiro, RJ, Brazil

Dear Editor,

Paracoccidioidomycosis is the main systemic mycosis in Latin America.^1^ It mainly affects rural residents due to soil manipulation and infection caused by inhalation of the fungi.^2^

The clinical classification of the disease comprehends a chronic form that occurs in adults, responsible for 80% of cases, and the acute/subacute juvenile form, representing 20% ​​of the cases.^3^

The authors report the case of an adult woman with a severe and atypical form of the disease, living in an urban area where several cases were described related to local environmental changes secondary to intense and prolonged soil movement during the construction of a metropolitan area highway that crosses urban centers in the state of Rio de Janeiro.^4^

A 61-year-old female patient, housewife, born and residing in the urban area of ​​the municipality of Nova Iguaçu, state of Rio de Janeiro, Brazil, presented with jaundice associated with abdominal pain for five months. She developed ascites, with weight loss and an increased number of lesions. She denied recent travel, comorbidities, smoking and alcohol consumption.

On physical examination, she had jaundice, a distended abdomen, hepatosplenomegaly with the right hepatic lobe located at three cm from the costal margin and a palpable spleen between the costal margin and the umbilicus. There was no palpable lymphadenopathy in the superficial chains. Dermatological examination showed multiple erythematous papules on the face, trunk, and limbs. In the cervical region, the papules were violaceous, with slight desquamation (Figure 1, Figure 2).

**Figure 1:**
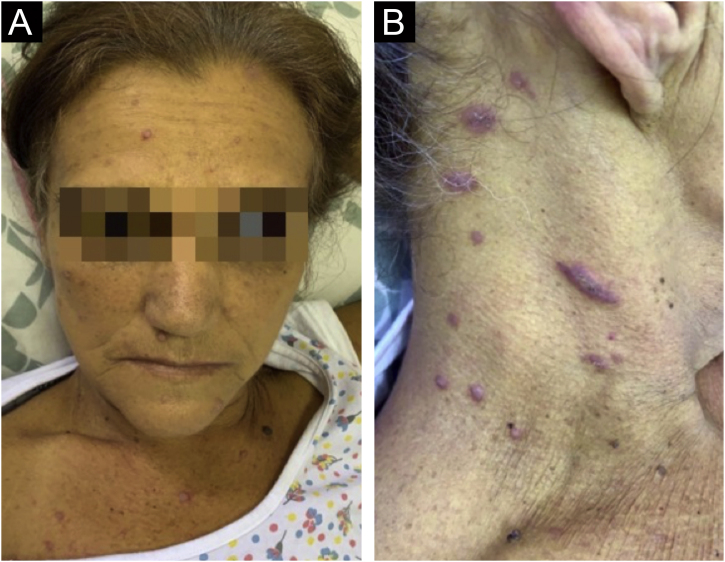
(A) Jaundice and erythematous papules on the face. (B) Violaceous papules with slight desquamation and a linear lesion in the cervical region.

**Figure 2:**
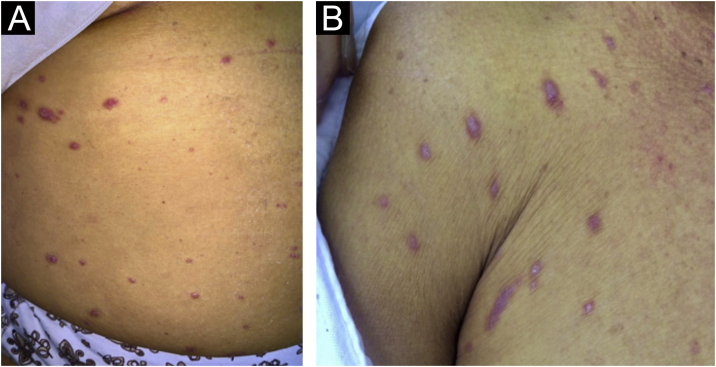
Erythematous papules on the abdomen (A) and axillary area (B).

The laboratory tests showed anemia, jaundice with a cholestatic pattern and a negative immunodiffusion test for paracoccidioidomycosis. The results of the tests of interest were as follows: hemoglobin 7.8 g/dL; hematocrit 23%; leukocyte count 11,900 μL (neutrophils 83%; lymphocytes 14%; monocytes 3%; and eosinophils 0%); platelets 700 × 10^3^ µL; aspartate aminotransferase 90 U/L; alanine aminotransferase 52 U/L; alkaline phosphatase 2,563 U/L; gamma-glutamyl transferase 233 U/L; total bilirubin 12.76 mg/dL; direct bilirubin 9.22 mg/dL; indirect bilirubin 3.54 mg/dL; albumin 2.5 g/dL; globulin 3.5 g/dL; prothrombin index 49%; activated partial thromboplastin time 44.2 seconds. Serological tests for hepatitis B and C, syphilis, and HIV were negative.

Computed tomography of the abdomen and chest showed ectasia of the intrahepatic bile ducts, pancreas with heterogeneous volume and density, bilateral pleural effusion and hepatosplenomegaly, in addition to several enlarged intra-abdominal lymph nodes (Fig. 3).

**Figure 3:**
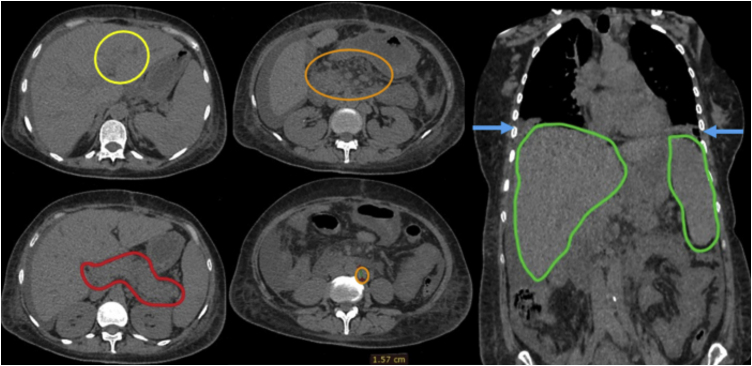
Computed tomography of the abdomen; yellow circle: intrahepatic bile duct ectasia; red circle: pancreas of heterogeneous volume and density; orange circles: enlarged intra-abdominal lymph nodes; blue arrows: bilateral pleural effusion; green circles: hepatosplenomegaly.

The main diagnostic hypotheses were hepatobiliary neoplasia, primary biliary cholangitis associated with lichen planus, and paracoccidioidomycosis.

Biopsies of the skin, liver and an intra-abdominal lymph node were performed. Histopathological examination of the skin disclosed chronic granulomatous inflammation, with a large number of neutrophils and multinucleated giant cells containing rounded structures in the cytoplasm. The Grocott staining showed spherical structures stained in brown, with multiple budding cells with a “marine pilot’s wheel” and “Mickey Mouse ears” appearance (Fig. 4).

**Figure 4:**
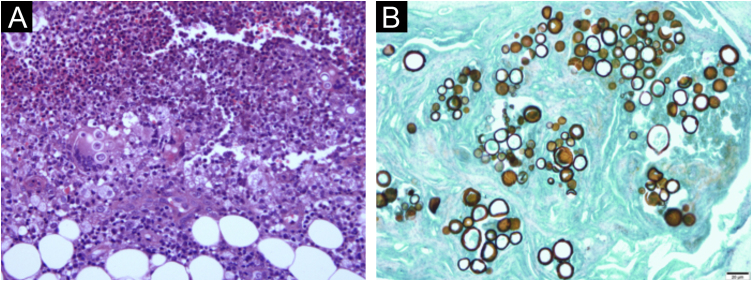
(A) Hematoxylin & eosin staining: chronic granulomatous inflammation process, with a large number of neutrophils and multinucleated giant cells containing rounded structures in the cytoplasm. (B) Grocott staining: brown spherical structures with multiple budding giving them a “marine pilot’s wheel” or “Mickey Mouse ears” appearance.

Histopathological examination of the liver and lymph node biopsy revealed chronic granulomatous inflammation with necrosis and the presence of fungal structures.

Histopathology confirmed the diagnosis of acute/subacute paracoccidioidomycosis and treatment with intravenous amphotericin B deoxycholate 1 mg/kg/day was started. However, the patient developed pancreatitis and acute renal failure, with the following laboratory tests: amylase 470 U/L; lipase 864 U/L; urea 118.8 mg/dL; creatinine 1.9 mg/dL. Therefore, the therapeutic regimen was modified to liposomal amphotericin B 5 mg/kg/day associated with meropenem 1 g IV 8/8 h and nutritional support measures, administration of intravenous fluids and amines. Blood cultures were performed and, although they did not show microorganisms, the patient developed a clinical picture compatible with refractory septic shock, due to pancreatic necrosis and died. An autopsy was not performed.

Among the mycoses, paracoccidioidomycosis is the main cause of death in immunocompetent patients. In Brazil, from 1998 to 2006, the disease was responsible for 50% of hospitalizations due to mycoses, with in-hospital mortality of 5%.^5^

Although the disease is rare in urban areas, this study describes the increase in the disease incidence after deforestation and the massive removal of land for the construction of a highway in the metropolitan area that crosses urban centers in the state of Rio de Janeiro. Before the highway construction, there were 1.29 cases for every million inhabitants in the region. After that, there was an increase to 8.25 cases per million inhabitants.^4^ The patient described in the present report was born and resided in Nova Iguaçu, where the construction was carried out.

Regarding the diagnostic hypotheses, it should be emphasized that, initially, the possibility of hepatocarcinoma was investigated, but liver, skin and lymph node biopsies showed the presence of paracoccidioidomycosis, correlating with the skin lesions and the clinical picture. However, before that primary biliary cirrhosis associated with lichen planus was included among the hypotheses, due to the lichenoid aspect of the lesions and the reports of the association of liver disease with this dermatosis.^6^, ^7^

In this case, the presence of jaundice was noteworthy, due to the severe hepatobiliary involvement, which is one of the systems most affected by the disease.^8^

The main causes of jaundice related to paracoccidioidomycosis are biliary obstruction due to lymph node enlargement, pancreatitis, intraluminal granulomatous lesion, and hepatitis. It should be emphasized that the first two above-mentioned causes were identified in this patient. In general, obstructive jaundice occurs when there is extrinsic compression of the common biliary duct caused by lymph node enlargement near the hepatic hilum. Pancreatitis, on the other hand, results from pancreatic duct drainage obstruction by regional ganglion compression.^9^

In conclusion, in endemic areas, it is important to consider paracoccidioidomycosis among the diagnostic hypotheses for jaundice and other signs and symptoms of cholangitis.^8^ Moreover, we must be alert to the emergence of cases in urban areas where environmental changes are taking place, especially with deforestation, which can affect the health-illness relationship.

## Financial support

None declared.

## Authors' contributions

Fernanda Altoé Stringuini: Design and planning of the study; drafting and editing of the manuscript; collection, analysis, and interpretation of data; effective participation in research orientation; intellectual participation in the propaedeutic and/or therapeutic conduct of the studied cases; critical review of the literature.

Priscila Oliveira Naback: Collection, analysis, and interpretation of data; intellectual participation in the propaedeutic and/or therapeutic conduct of the studied cases.

Luciana Ferreira Araújo: Approval of the final version of the manuscript; collection, analysis, and interpretation of data; effective participation in research orientation; intellectual participation in the propaedeutic and/or therapeutic conduct of the studied cases; critical review of the manuscript.

Ricardo Barbosa Lima: Approval of the final version of the manuscript; design and planning of the study; collection, analysis, and interpretation of data; effective participation in research orientation; critical review of the literature; critical review of the manuscript.

Carlos José Martins: Approval of the final version of the manuscript; design and planning of the study; effective participation in research orientation; critical review of the manuscript.

## Conflicts of interest

None declared.
